# Neuroimaging Modalities in Alzheimer’s Disease: Diagnosis and Clinical Features

**DOI:** 10.3390/ijms23116079

**Published:** 2022-05-28

**Authors:** JunHyun Kim, Minhong Jeong, Wesley R. Stiles, Hak Soo Choi

**Affiliations:** 1 Gordon Center for Medical Imaging, Department of Radiology, Massachusetts General Hospital and Harvard Medical School, Boston, MA 02114, USA; oaksa03@pusan.ac.kr (J.K.); jeong.minhong13@gmail.com (M.J.); wstiles2@mgh.harvard.edu (W.R.S.); 2 Department of Cogno-Mechatronics Engineering, Pusan National University, Busan 46241, Korea

**Keywords:** neuroimaging, multimodal imaging, brain imaging, Alzheimer’s disease, early diagnosis, preclinical biomarkers

## Abstract

Alzheimer’s disease (AD) is a neurodegenerative disease causing progressive cognitive decline until eventual death. AD affects millions of individuals worldwide in the absence of effective treatment options, and its clinical causes are still uncertain. The onset of dementia symptoms indicates severe neurodegeneration has already taken place. Therefore, AD diagnosis at an early stage is essential as it results in more effective therapy to slow its progression. The current clinical diagnosis of AD relies on mental examinations and brain imaging to determine whether patients meet diagnostic criteria, and biomedical research focuses on finding associated biomarkers by using neuroimaging techniques. Multiple clinical brain imaging modalities emerged as potential techniques to study AD, showing a range of capacity in their preciseness to identify the disease. This review presents the advantages and limitations of brain imaging modalities for AD diagnosis and discusses their clinical value.

## 1. Introduction

**Figure 1:**
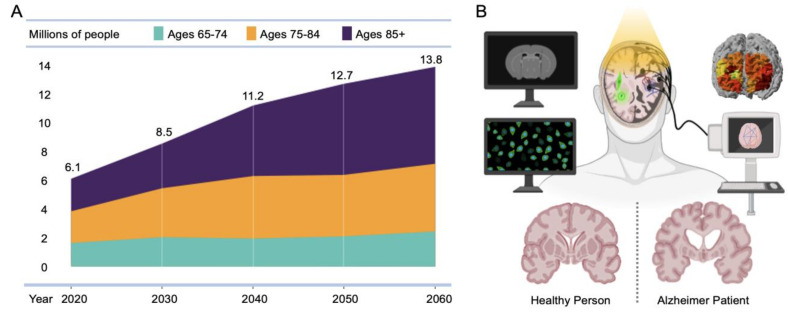
AD population and diagnostic neuroimaging modalities. (**A**) Projected number of people aged over 65 in the U.S. population with Alzheimer’s dementia from 2020 to 2060. Adapted from [1] with permission from John Wiley and Sons. (**B**) Multimodal neuroimaging modalities to diagnose AD patients.

Dementia is a set of symptoms of impaired cognitive functioning that can develop in the damaged brain [1]. There are several different causative diseases from which dementia can arise, with Alzheimer’s Disease (AD) being the most common cause, entailing severe forms of cognitive dysfunction. The most significant risk factor for AD is aging, and cases are expected to continuously rise year by year, remaining one of the most critical challenges associated with the aging population (Figure 1) [1]. Deviating from the normal path of aging, Alzheimer’s dementia leads to notable brain damage, followed by subsequent functional disorders. The exact causes of this brain degeneration have yet to be clearly explained, but the leading hypothesis regarding its pathology involves the development of abnormal proteins between neurons. Currently, diagnosis requires the presence of extracellular senile plaques and intracellular neurofibrillary tangles that show stereotypical distribution patterns spreading throughout the brain [2]. These senile plaques are known to contain neurotoxic amyloid-β (Aβ), and neurofibrillary tangles are comprised of hyperphosphorylated tau protein, whose accumulation ultimately results in the loss of synapses and neurons in the affected regions [3]. These core neuropathologies are known to spread in spatiotemporal stages that lead to neuronal degeneration in the brain [4], resulting in the appearance of clinical symptoms of dementia such as memory loss and behavioral changes. While aging is the most critical factor, carrying genetic risk factors, such as mutations in presenilin genes, amyloid precursor protein (APP), or the apolipoprotein E gene (APOE), also increases the risk of developing plaques and tangles [3].

AD is characterized by a long preclinical stage where pathological changes can start years before clinical symptoms manifest. Having these pathological markers, however, does not always mean that patients will develop severe cases of dementia, and patients in the mild cognitive impairment (MCI) stage may not meet the criteria for clinical diagnosis [2]. The difficulty in distinguishing between asymptomatic and non-AD MCI groups makes diagnosis trickier, which may explain the challenges observed in clinical trials [5,6].

Brain imaging modalities provide time-sensitive information and have the potential to enable early diagnosis, even before the cascade of neurodegenerative processes begins, which can classify patients into different stages. Showing cognitive impairment likely indicates advanced disease progression, and various imaging methods have been utilized to delineate the characteristics of AD-related groups (Table 1). The diagnostic criteria of AD include not only measuring the changes in the white matter, gray matter, and blood vessels in the brain, but also finding synaptic dysfunctions caused by the accumulated Aβ and tau proteins, both of which rely heavily on neuroimaging. However, each diagnostic methodology may lead to some discrepant conclusions, making it difficult to differentiate clinical cases and determine staging. A comprehensive analysis of the available brain imaging modalities is thus needed. Herein, we review multiple neuroimaging techniques that can be utilized in AD research and discuss their strengths and limitations as well as their reliability as clinically informative tools for AD research. 

## 2. Neuroimaging Modalities in AD

### 2.1. Structural MRI

#### 2.1.1. Basic Principles

**Figure 2:**
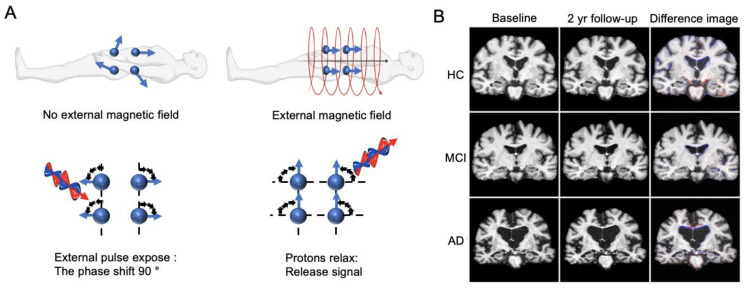
MR imaging. (**A**) Principles of MR imaging. (**B**) MR images of healthy control (HC) subject, mild cognitive impairment (MCI) subject who converted to AD after three years, and an AD patient. Adapted from [41] with permission from Springer Nature.

Magnetic resonance (MR) imaging is one of the most common imaging techniques employed to visualize the internal organs in the body and can also be used to image the anatomy of the brain. MR imaging uses strong magnets and low-energy radiofrequency signals to gather information from atomic nuclei within the body [31,32]. Hydrogen, a common atom present in every molecule in organic compounds, is highly suitable for imaging because of its abundance in the human body. In the context of imaging, hydrogen nuclei can be regarded as small bar magnets with north and south poles that spin on their axes. Figure 2A illustrates the magnetic moments of hydrogen in the body. When a human is exposed to a strong external magnetic field, the hydrogen nuclei align parallel or anti-parallel to the external magnetic field. Without a magnetic field, the magnetic moments of the nuclei are distributed at random, and thus the net magnetization factor is zero. Simply aligning hydrogen nuclei with an external magnetic field is not enough to produce a signal for imaging due to their static state. To obtain a signal, hydrogen nuclei need to be exposed to a radiofrequency energy pulse (resonance frequency). When exposed, the protons in hydrogen nuclei attain a high-level energy state, and the net magnetization changes to 90°. The MRI system measures the alternating current from the receiver coil, which occurs from the rotating transverse magnetization of hydrogen nuclei. With structural changes contributed by dendritic and neuronal losses in the AD brain [33], MRI utilizes the fact that hydrogen nuclei within the atrophied regions cannot be detected clearly, thus enabling the assessment of disease progression.

#### 2.1.2. Applications in AD Diagnosis

The pathological nature of AD is expressed as structural changes in the brain, including anatomical location, cortical thickness, volumetry, and other morphological characteristics [34]. Structural MRI scanning is widely used to measure changes in brain morphometry related to the loss of neurons, synapses, and dendritic de-arborization in AD progression over time [35,36]. MRI results show that grey matter atrophy starts even in the preclinical stages of AD when the brain is cognitively normal, with regional cortical thinning appearing prior to the onset of AD dementia [37]. The earliest sites of atrophy typically occur at the entorhinal cortex in the medial temporal lobe, followed by the hippocampus and adjacent structures [33]. Due to its early volumetric reduction, the medial temporal region, including the hippocampal area, has been the main target of analysis [8,9,38], with the hippocampal volume being a better indicator of AD neuropathology in a post-mortem MRI scanning study of individuals who remained non-demented but had memory impairment [8]. 

The anatomical characteristics of AD are not limited to volumetric reductions in the whole brain or hippocampal area. Other measures such as cortical thickness or ventricular enlargement in correlation with the accumulation of senile plaques and cortical neurofibrillary tangles also represent a quantitative index for MRI assessment [10,39,40]. The progressive atrophy is depicted in Figure 2B, where the increased atrophy in the medial temporal lobe and the enlarged ventricles are visually apparent in the different images. The ability to quantitatively measure these morphological features using MRI has led to a surge of methodological development in predicting and classifying AD [41]. 

#### 2.1.3. Pros and Cons of Using MRI in AD

The major strength of MRI is its widespread availability to be used in AD research. MR images provide excellent anatomical detail, and hippocampal atrophy measured on a high-resolution T1-weighted MRI serves as a critical criterion for the clinical diagnosis of AD. Manually segmented images and automated algorithms provide reliable classification results with high diagnostic accuracy that correlate well with the underlying pathology [36]. 

Although measuring atrophy possesses clinical strength, structural MRI lacks the molecular specificity to directly determine the neural source of volume or thickness loss and whether it is cell loss or dendritic and synaptic loss [34]. Cerebral atrophy, hippocampal atrophy, or ventricular dilation are often present to some degree in normal aging and other diseases, which may be caused by factors other than the progression of neuronal loss. Moreover, structural MRI cannot assess functional changes, so the combination with other measurements may enhance the accuracy of AD detection.

### 2.2. FDG-PET

#### 2.2.1. Basic Principles

**Figure 3:**
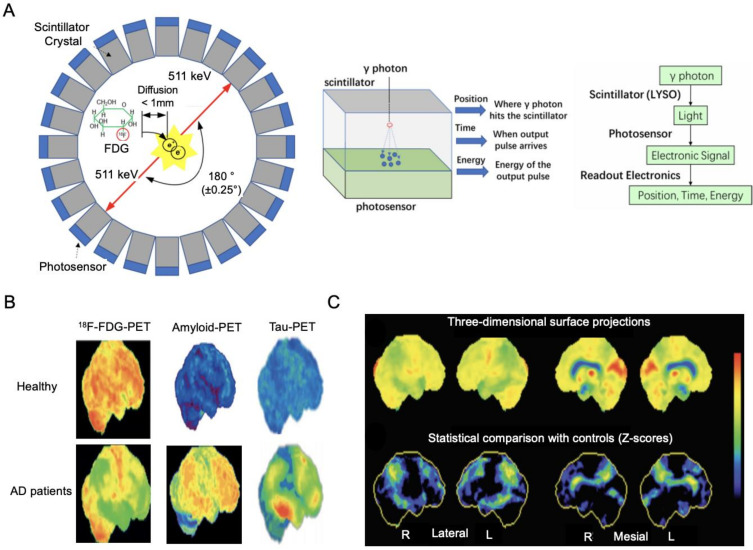
FDG-PET imaging. (**A**) Structure of PET detector and detection flow chart. Adapted from [45] with permission from MDPI. (**B**) Comparison of ^18^F-FDG-PET, amyloid-PET, and tau-PET images from healthy controls (top row) and individuals with late-onset AD (bottom row). Adapted from [44] with permission from Elsevier. (**C**) A 3D surface projection of ^18^F-FDG PET findings in AD (top row) and a statistical comparison with a healthy control population; green or yellow shows high deviation and black or blue shows no or low deviation (bottom row). Adapted from [53] with permission from Elsevier.

Positron emission tomography (PET) imaging measures metabolic changes using different radioactive tracers depending on the intended target [42]. PET imaging is a coincident detection of gamma rays released from annihilation events of positrons from the radiotracer. Here, the radioactive tracers are analogous to common biological molecules such as glucose, peptides, and proteins, in which a radioisotope is used to substitute one of the atoms of the tracer [43,44]. When a radioactive tracer is injected into the subject, the tracer arrives at the targeted organs or tissues through the circulatory system and participates in metabolic processes [42]. As shown in Figure 3A, the radioisotopes in the tracer decay due to their instability, and during this decay process positrons are ejected and collide with the electrons of an adjacent atom, resulting in an annihilation process [45]. The annihilation produces two 511 keV gamma rays with a separation of approximately 180 degrees, which are absorbed by scintillation crystals and converted into low-energy visible photons [45]. A photosensor then converts the light signals into electrical signals. A detector is formed from scintillators, detectors, and readout electronics that record three metrics: the time when the gamma ray hits the detector, the position where the gamma ray hits, and the energy of the gamma ray [46]. These electronic signals are then processed to generate an image through corrections and reconstructions [45]. Depending on the radiotracer, PET imaging varies in its ability to map different signatures, including brain metabolic activity, amyloid burden, or tau-tracer retention (Figure 3B). PET is used as a diagnostic tool to compare the scans of AD patient and non-patient groups to see the differences in uptake patterns [44].

#### 2.2.2. Applications in AD Diagnosis

While structural imaging captures the downstream pathological changes, it is not appropriate for reflecting changes that precede protein deposition. With evidence that asymptomatic individuals with a genetic risk of AD show metabolic changes that precede atrophy [47], there is a presumption that there are functional biomarkers that can be detected prior to the distinct protein signatures associated with progressed AD [48]. According to a meta-analysis [49], PET imaging with ^18^F-fluorodeoxyglucose (FDG) performs better than structural MR imaging in predicting conversion to AD dementia and, therefore, is more useful for early diagnosis.

The biomarker of neurodegeneration detected by FDG-PET in dementia patients is brain hypometabolism [44], and the changes in FDG uptake in AD patients correlate with cognitive decline [50]. Although a decrease in glucose metabolism is also found in normal aging [51,52], the dementia caused by AD carries different metabolic changes. Comparing patients with AD and healthy controls in their regional cerebral glucose metabolism, the patients with AD show a much greater decline in glucose metabolism and had additional reductions over 1 year compared to the healthy controls [11]. The significant deviations in cortical metabolism in AD patients compared to healthy controls are also illustrated in Figure 3C [53]. Longitudinal studies show that glucose metabolic reductions can precede clinical diagnosis and predict cognitive decline years before symptomatic onset [12,54], which makes glucose hypometabolism a viable predictor for the disease. 

The diagnostic ability of FDG-PET in AD has been the subject of many studies. It was once shown that FDG-PET can achieve 90% sensitivity in identifying AD [55], but later studies disproved this result, showing that patients with MCI who converted to AD could be diagnosed using the pooled estimates of FDG-PET with 78.7% sensitivity (95% CI, 68.7–86.6%) and 74.0% specificity (95% CI, 67.0–80.3%) [56]. It has been validated that the use of deep learning algorithms can lead to an improved predictive ability prior to final diagnosis, with one model achieving up to 82% sensitivity and 100% specificity [57]. A recent review based on meta-analyses of articles regarding the identification of AD patients among healthy individuals resulted in pooled sensitivities up to 96%, with specificities up to 90% [58]. This data-driven approach needs consistency in its feature extraction. Therefore, the need for a reliable segmentation of reference regions in FDG-PET studies has been raised [59].

#### 2.2.3. Pros and Cons of Using FDG-PET in AD

Brain dysfunction measured by FDG-PET can be diagnostically superior to simple volumetric measures, as FDG-PET can capture neurodegeneration in AD earlier than MRI [44]. Although its high resolution adds value to its relatively high reliability in identifying the early stages of AD, PET scanners have limited availability due to their high cost. Moreover, FDG-PET requires an injection of radiolabeled tracers, which is considered more invasive than other neuroimaging modalities. Nevertheless, FDG-PET can provide a more detailed diagnosis of brain cognitive metabolism and synaptic dysfunction by quantifying toxic Aβ and tau proteins in the brains of AD patients, which drive healthy neurons into the diseased state [60,61,62]. Moreover, hypometabolism is a result of neurodegeneration and therefore might not be suitable to detect the signs of AD in the earliest stages before neuronal loss occurs [63].

### 2.3. Functional MRI

#### 2.3.1. Basic Principles

**Figure 4:**
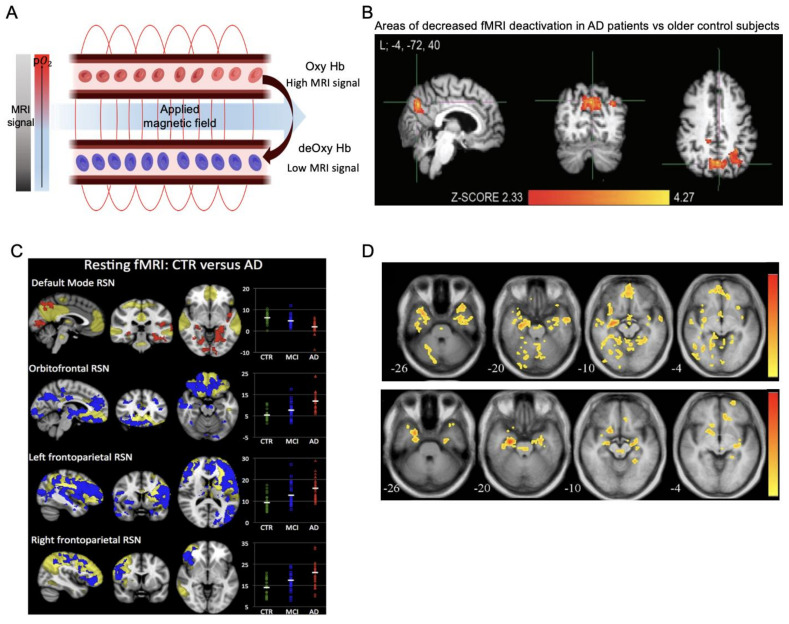
fMRI imaging. (**A**) BOLD effect in MRI. (**B**) Areas of decreased fMRI task-induced deactivation in posteromedial cortical areas in patients with AD compared to older controls. Adapted from [67] with permission from Hindawi. (**C**) Spatial maps of resting-state networks (RSNs, transparent yellow) overlaid with clusters showing significantly lower (red) or higher (blue) functional connectivity in AD compared to control subjects (CTR). Adapted from [76] with permission from BMJ Publishing Group Ltd. (**D**) Right hippocampal connectivity maps in the healthy age-matched control group (top row) and AD group (bottom row). Adapted from [77] with permission from Elsevier.

Functional MRI methods image neural activation indirectly by detecting changes in the hemodynamics of brain vessels. The hemodynamic response is one method to confirm activation in brain regions depending on the amount of oxy- and deoxy-hemoglobin present. The blood oxygenation level-dependent (BOLD) technique relies on the paramagnetic properties of deoxy-hemoglobin in the blood [64] and on intact neurovascular coupling. That is, the increase in oxygen consumption in the brain that follows a surge of neuronal activity is normally associated with enhanced cerebral blood flow (CBF) and blood volume (CBV) to the activated area [65], with the converse taking place during decreases in neuronal activity. The magnetic susceptibility of the deoxygenated blood is low, causing the MRI signal to decay faster [66]. Oxygenated blood flow in the activated regions of the cortex increases the MRI signal with a more magnetically uniform environment, which is then recorded in the fMRI (Figure 4A). fMRI images examine the correlation between the BOLD signal and brain regions by comparing the cognitive condition to a control condition or by investigating functional connectivity during the resting state [33]. Considering the cognitive impairment in the course of AD, fMRI techniques can monitor brain dysfunction related to AD.

#### 2.3.2. Applications in AD Diagnosis

Early AD research with fMRI focused on task-induced brain activation differences in AD patients compared to normal elderly individuals, and these task-activated paradigm studies revealed several AD-like alterations in the brain that correlate with functional deficits. Figure 4B demonstrates cortical deactivation during the processing of repeated face–name pairs, an example of an fMRI image typically acquired in AD patients compared to normal controls [67]. With memory impairment being the most critical sign of AD during the symptomatic phase, the most common findings have reported decreased activation in the hippocampus or medial temporal lobe during memory performance tasks in MCI or AD patients [68,69]. The amount of activation detected by fMRI in AD patients varied greatly. However, there is evidence of an increased activation phase during the prodromal stage of AD [69]. Longitudinal fMRI assessments in elderly individuals revealed that subjects who showed the highest baseline hippocampal activations had the most rapid declines in hippocampal activation [70] as well as amyloid and tau-related increases in the brain and activated downstream pathways [19,21]. Discrepant results may likely be attributed to differences in specific paradigm demands, stages of impairment, or behavioral performance. However, both hyper- and hypo-activation have been found in AD cohorts [16,17,20], indicating a nonlinear effect of fMRI activation patterns along the AD continuum from the initial hyperactivity of cognitive regions in preclinical or mild AD cases to the distinct loss of activation in the later stages. Data also suggest the possibilities of preclinical biomarkers that can be used for early diagnosis. AD pathology in the asymptomatic stages also correlated with aberrant fMRI activity [14,18,21,71], which is different from the functional decline resulting from normal aging.

Resting-state fMRI (rs-fMRI) examines the synchronous and spontaneous BOLD signal fluctuations in the absence of any experimental tasks during scanning [72]. Analyses largely focus on the default-mode network (DMN), which mainly includes the posterior cingulate cortex (PCC), precuneus, and medial temporal lobes, representing disruptive changes in metabolism and structure in AD [73]. Since severely impaired patients may be too limited to perform cognitive tasks during scanning, rs-fMRI may be more feasible to monitor disease progression in later stages. Studies have found alterations in this large-scale functional network as the disease progresses, where AD patients display deficient network connectivity compared to normal controls [23,74,75]. Figure 4C illustrates the difference map between control subjects and AD patients, where the controls have greater functional connectivity in the medial posterior cortex and extending to the hippocampal area [76]. Figure 4D also shows differential resting state hippocampal connectivity, where healthy controls show greater connectivity to multiple brain regions (top row) compared to the AD group (bottom row) [77]. The lower functional connectivity within the default mode network in AD patients was clinically relevant to cognitive dysfunction [24]. The connectivity showed a nonlinear pattern that also tracked with amyloid and tau deposition [26], agreeing with the findings from task-based fMRI studies that AD is characterized by both increased and decreased activation. Aggregated data of fMRI findings in AD have led to a surge in methodological research regarding patient classification. Novel methods of feature extraction with fMRI images have been proposed. For example, Guo et al. [78] showed an accuracy, specificity, and sensitivity of 91.6%, 90.5%, and 93.5%, respectively, by combining brain region features and subgraph features. Amini et al. [79] utilized multiple machine learning methods for identifying disease severity, which achieved an accuracy and sensitivity up to 91.7% and 98.1%, respectively. The automated classification and diagnosis of AD using the rs-fMRI dataset [80,81,82] also provide a new approach to improve diagnostic accuracy and capture brain alterations.

#### 2.3.3. Pros and Cons of Using fMRI in AD

The noninvasive nature of fMRI systems enhances the usability along with other imaging systems, such as MRI, and the spatial resolution tops other imaging systems in demonstrating activated brain networks when subjects undertake tasks. The excellently formulated graphics are, however, the reflection of surrogate signals that measure mass neuronal activity, which is easily subject to many internal and external disruptions. When there is significant motion of the subject or variation in metabolic blood levels, the magnitude of the BOLD response is affected, and its reliability in localizing activity decreases. The application of data analysis techniques is another important area for further development. The recent use of fMRI imaging for AD recognition has been extended to machine learning and deep learning techniques, but different algorithms and analysis methods lead to many controversial findings. The major drawback of AD imaging using fMRI, and many other imaging systems in general, is that functional changes are not AD-specific. For example, reduced DMN connectivity is found in patients with other psychiatric disorders or diseases, thus requiring a more innovative approach to draw conclusions.

### 2.4. fNIRS

#### 2.4.1. Basic Principles

**Figure 5:**
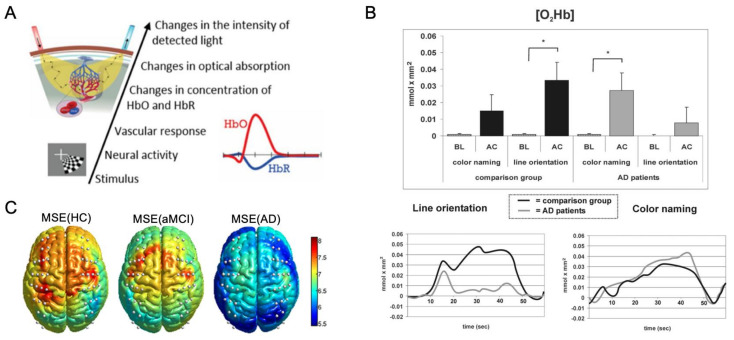
fNIRS imaging. (**A**) Schematic diagram of functional near-infrared spectroscopy (fNIRS) system. Adapted from [85] with permission from Elsevier. (**B**) Average increase in O2Hb during the baseline (BL) and activation phase (AC) for AD and comparison groups in the line orientation task. * *P* <0.05. Adapted from [86] with permission from Elsevier. (**C**) Brain activation represented by spatial maps of multiscale entropy (MSE) analysis in HC, aMCI, and AD groups. Adapted from [89] with permission from OSA Publishing Group.

Functional near-infrared spectroscopy (fNIRS) is a noninvasive optical imaging modality that monitors the hemodynamics of blood vessels, similar to fMRI, which uses light from the NIR window (700–900 nm). Since NIR light is less scattered and absorbed by few biological chromophores, it easily passes through biological tissues [83] and can penetrate the skull, being absorbed by oxy-hemoglobin (HbO) and deoxy-hemoglobin (HbR) with different absorption spectra [84]. The changes in oxygenation–deoxygenation states are calculated by the amount of transmitted light through the tissue (Figure 5A) using the modified Beer–Lambert Law, and the summation reflects the total blood volume during brain activity [85]. As more blood flows to the activated brain regions, NIRS can be used to detect neural dysfunction when brain shrinkage occurs by neurodegeneration.

#### 2.4.2. Applications in AD Diagnosis

fNIRS studies are generally similar to fMRI studies in that they measure the intensity and pattern of neuronal activities and highlight the activated regions underlying a performed task. The focus of using fNIRS in AD is typically to investigate the overall functional activation, as depicted in Figure 5B, which tracks the concentration changes in oxyhemoglobin and deoxyhemoglobin in AD patients compared to controls during a line orientation task, where AD patients showed difficulty with only a marginal metabolic increase [86]. Consistent with fMRI findings, the cognitive functioning in AD detected by fNIRS observes decreased oxygenation levels compared to non-AD groups.

The neurofunctional deficit in AD, indicated by a reduced oxygenation level, is characterized by various features. It was, for instance, associated with hemispheric asymmetry, with AD patients demonstrating the loss of lateralized activation in a verbal task, but involving global activation in the right hemispheric regions, which was not observed in the controls [87]. A multiscale entropy (MSE) analysis pointed out different signal complexity in its relevance to clinical symptoms of AD [88]. Such abnormal patterns of hemodynamic response open up the possibility of tracking the progression and early detection, as the patterns are not observed uniformly within varying degrees of AD [13]. 

Functional connectivity analysis can also be performed for the characterization of intrinsic brain activity using fNIRS. As shown in Figure 5C, findings are similar to rs-fMRI, with AD patients showing reduced signal complexity in most brain regions [89]. Moreover, the synchronization of fNIRS signals is reduced in mild AD subjects compared to normal aging controls, with a loss of regularity within the brain network with disease progression [90]. Though these typical statistical analyses based on the region of interest (ROI) are suitable for detecting differences between groups, they cannot provide an individual diagnosis. The development of novel classification methods is also being studied, for example, utilizing the change in oxygenated hemoglobin at specific time points to identify the patient group by a convolutional neural network (CNN) [91], promising that future studies could be improved by using advanced deep learning techniques.

#### 2.4.3. Pros and Cons of Using fNIRS in AD

Compared to other systems, fNIRS is a novel modality with the benefits of being non-invasive, safe, and relatively low-cost. Frequent brain scanning is possible with relatively good temporal resolution, and its portability and robustness to motion enable monitoring during action [90]. Its feasibility with AD assessment has been proven, as NIRS revealed activation deficits in the parietal lobes of AD patients during cognitive task performances compared to controls [86], which opens the possibility of NIRS becoming an early detection method in the future. 

However, the abnormal plaques and neurofibrillary tangles observed in patients’ brains are said to be responsible for the disconnection of neuronal networks. Functional connectivity, which reveals the neuronal communication network, is therefore more relevant than brain activation, which only demonstrates hemoglobin changes in individual brain regions, and should be considered in future studies. More importantly, as other cognitive disabilities yield similar findings [92], fNIRS may not yet be ready to be used as an AD-specific diagnostic tool. The non-established standard of data processing also makes it challenging to provide individual diagnosis results, leaving more room for improvement in clinical applications.

### 2.5. EEG

#### 2.5.1. Basic Principles

**Figure 6:**
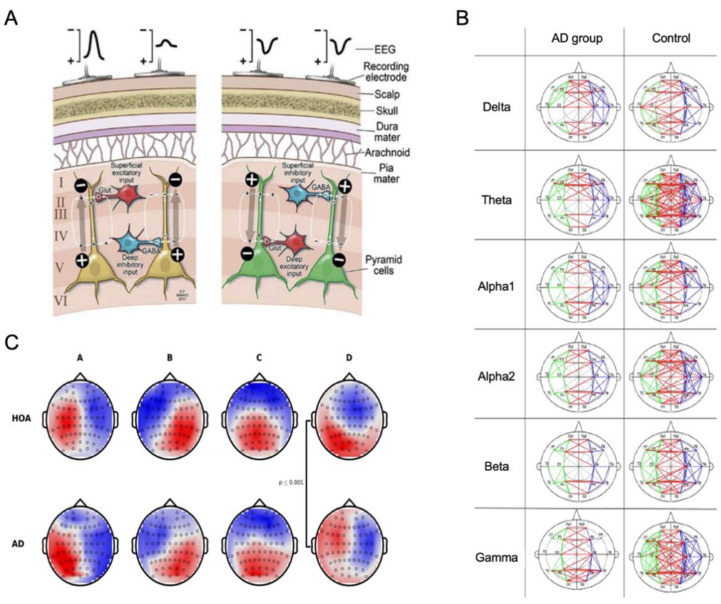
EEG imaging. (**A**) The mechanism of capturing electroencephalogram (EEG) signals and the generation of EEG network oscillations. Adapted from [94] with permission from Elsevier. (**B**) The functional networks of AD and control groups in six frequency bands. Adapted from [102] with permission from AIP Publishing. (**C**) Microstate topographies of healthy older adults (HOA, top row) and AD (bottom row) cohorts. Adapted from [30] with permission from Springer Nature.

As shown in Figure 6A, electroencephalography (EEG) can record the joint electrical activity generated by the brain between millions of active neurons using electrodes at the surface of the scalp or intracranially [93,94]. The EEG activity mainly reflects the more or less synchronous activation of a large population of neurons and, more precisely, their postsynaptic activity. The intracranial mean measure of this postsynaptic activity is called the local field potential. If a large population of neurons are spatially aligned and have synchronous activity, the resulting superimposed electrical field will be detected by electrodes at the scalp surface. This situation is often encountered for cortical pyramidal neurons since they are oriented perpendicularly to the cortical surface, meaning their activity is most likely to be detected by EEG [94,95]. EEG tracks the electrical activity and compares the patterns and connectivity to find any abnormalities present in the patient group. 

#### 2.5.2. Applications in AD Diagnosis

The electrical activity measured with EEG reflects functional changes in the cerebral cortex, and abnormalities in this activity can be used to detect the functional deficits caused by neurodegeneration in AD. While the signal recording can be conducted in diverse conditions, the resting-state EEG, in the absence of any cognitive activity, is easier to perform with AD patients [96], as it avoids any patient discomfort or possibilities of not being able to complete the required tasks. Commonly reported effects of AD on EEG signals include gradual complexity loss, connectivity alterations, and changes in microstate complexity. To diagnose AD using EEG, many researchers use various frequencies to discriminate healthy persons from AD patients. For example, the 0.1–4 Hz (delta band) range is used to detect the thalamus region and cortex, 4–8 Hz (theta band) to detect cognition, behavior, and memory in the hippocampus, 8–12 Hz (alpha band) to detect the thalamus region, 12–30 Hz (beta band) is associated with the motor cortex and anxious thinking, and 30–100 Hz (gamma band) is used to detect various regions, including the premotor, parietal, and temporal cortical regions [97]. Spectral analysis studies report that AD induces increased activity in the delta and theta frequency bands as well as decreased activity in the alpha and beta bands [98,99,100,101].

A slowing of brain oscillatory activity, indicated by a higher power in low-frequency bands and lower power in high-frequency bands, has been reported in non-demented amyloid-positive patients [28], suggesting synaptic dysfunction starts from the early stages of AD. The presence of genetic risk factors can also predict AD progression before its clinical onset, as reported by signal complexity loss along the AD continuum with differing complex patterns between APOE carriers and non-carriers [29]. Analyses of EEG signals have also revealed disruptions in functional connectivity. Wang et al. [102] reported decreased pairwise coherence in AD, along with decreased mean network connectivity in AD patients for each frequency band (Figure 6B). The disruption in functional connectivity was also demonstrated by aberrant lagged phase synchronization in AD, which correlated with cognitive decline [98]. 

Among EEG analyses, the microstate analysis is thought to be a viable measurement for exploring changes in brain dynamics within a millisecond scale that may signal impaired cognitive functioning in AD [30]. Instantaneous topographic maps are depicted in Figure 6C, showing that the AD cohort has altered topography of microstate class D, the frontoparietal network related to the attention and working memory cognitive domains [30]. 

EEG biomarkers would expand upon the existing collection of AD pathology biomarkers [100] and could be used to distinguish AD cohorts in different stages. With mild probable AD subjects, higher spectral power was found in the alpha band compared to healthy controls during memory tasks [103], and Gaubert et al. [104] reported a non-linear relationship between the amyloid burden and EEG metrics. These results point to a pattern of EEG changes that appears to reflect the progression of the disease, starting with moderate increases in power at the slow frequencies and, in the later stages of progression, a decrease in the faster frequencies.

Data-driven methods also contribute to exploring the utility of EEG for the assessment of cognitive decline due to AD. A novel distance measure between the probability distribution function of theta and alpha power resulted in a significant improvement in classification accuracy [100]. Specifically, by using epoch-based entropy and bump modeling, Houmani et al. [99] reported a classification accuracy of 91.6% when discriminating subjective cognitive impairment patients from possible AD patients.

#### 2.5.3. Pros and Cons of Using EEG in AD

EEG can provide neuronal information within a millisecond timescale. This high temporal resolution is the most significant advantage of this technique compared to other imaging techniques such as MRI or PET. However, the distance between the electrodes and the actual source of neuronal activity is a vital drawback of EEG measurements since it creates low-pass filtering on the source signal. Thus, the spatial resolution limitation can become a problem when trying to precisely describe neuronal processes. Regarding EEG processing, the manual selection of EEG epochs often introduces human biases, and multiple unestablished processing methods may lead to differing classification results. Although EEG can provide insight into possible spatiotemporal distribution patterns of disease progression, subtle EEG abnormalities are hard to detect in the early stages of AD and are not distinct from the markers of other neurodegenerative diseases (Table 2).

## 3. New Approaches of Neuroimaging in AD Research

### 3.1. Multimodal Imaging 

The clinical manifestations of AD vary by symptomatic stages and the presence of certain pathological markers. Different measurements, such as spinal fluid assessments, cognitive tests, and imaging techniques, are combined for a clear examination and to conclude the possibility of AD [115]. Neuroimaging studies of AD, therefore, cannot rely on a sole system to evaluate neurodegeneration but require data from multiple systems. Multimodality is the combination of “anatomy + activity + connectivity” analysis in the brain [115], and existing studies are often conducted in a paradigm where anatomical and functional screening are both used. 

**Figure 7:**
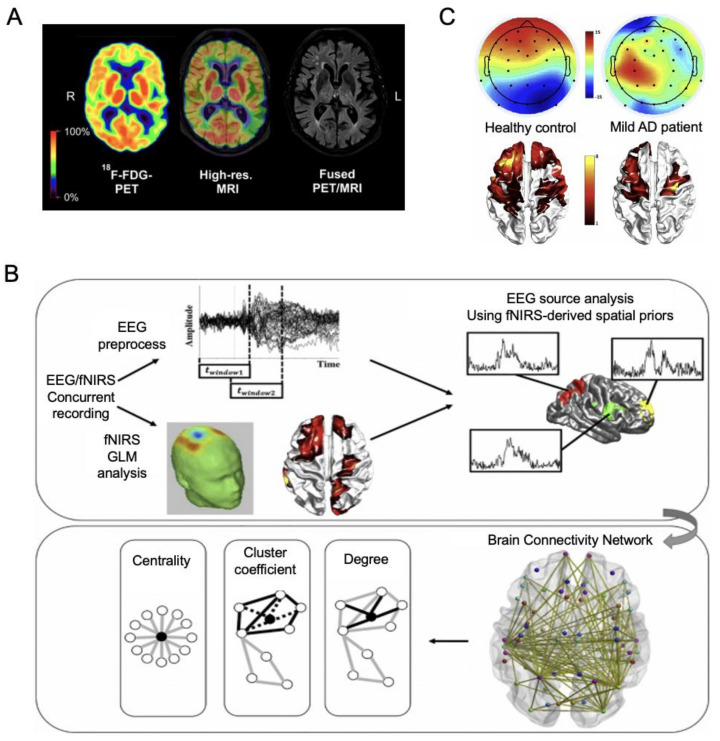
Multimodal Imaging. (**A**) Simultaneous acquisition of PET and MRI images in an AD patient. Adapted from [116] with permission from Society Nuclear Medicine and Molecular Imaging. (**B**) Concurrent recording of EEG and fNIRS and the resulting representative EEG topographies and fNIRS activation maps for the healthy subject and mild AD patient. (**C**) EEG and fNIRS: Dynamic cortical connectivity alterations associated with AD. Adapted from [117] with permission from Elsevier.

Commonly seen in fMRI studies, prior assessment through MRI or PET systems gives primary information on the patient groups, making between-group comparisons possible. The findings report the presence of group-specific features of neurodegenerative signal changes that might represent a biomarker of certain AD stages or identify the severity of AD. Figure 7A shows a novel approach of AD assessments with both high-resolution MRI and ^18^F-FDG-PET, in which the simultaneous screening may allow for more precise staging of the disease by detecting both molecular and functional abnormalities [116]. 

Meanwhile, each imaging system carries mechanistic limitations in deriving clinical conclusions. In this case, researchers may adopt a multisystem approach to reinforce each system in its diagnostic reliability. In EEG, insufficient spatial resolution and instability to noise lower its effectiveness, but the fusion with fNIRS systems has been proven useful in understanding the dynamics of brain activity [117]. Figure 7B is an example flowchart of integrative analysis in the EEG-fNIRS technique where EEG source localization is based on spatial priors from fNIRS, which shows the EEG signal topographies and fNIRS activation maps between a healthy subject and an AD patient [117].

Recently, several publicly available neuroimaging databases allowed combined analyses from multiple data modalities. Studies report the effectiveness of employing the hybrid model, for which new statistical methods or algorithms are used [118], and these approaches enable the simultaneous quantification of clinical features and brain activation, making their relationship clearer. For example, machine learning techniques in EEG-fNIRS imaging could compensate for the shortcomings of each modality to assess cognitive abilities and patient classification [118,119], and the use of novel deep learning processing frameworks has been confirmed to be effective in the discrimination of patient groups and whether the controls and MCI patients will convert to AD in the following years [120]. While there is an indication that these multimodal data are not always linearly related, the newly proposed non-linear graph fusion technique achieved a high classification performance [121], which validated the benefit of using complementary information for AD classification. The differential approaches of using automated classification can assist clinical decisions in neuroimaging diagnosis.

### 3.2. Noninvasive Fluorescence Imaging

Optical fluorescence imaging is the most powerful modality to diagnose AD pathology and neural networking by confirming the fluorescence of Aβ and/or tau proteins and other AD-associated proteins. Fluorescence imaging uses endogenous and/or exogenous fluorophores that irradiate light upon laser excitation. Particularly, NIR fluorescence imaging (650–1000 nm for NIR-I and 1000–1700 nm for NIR-II) can display high sensitivity and specificity for the real-time imaging of biological systems due to minimal autofluorescence, low light scattering, and low light absorption in neighboring tissues [122,123,124,125,126,127]. This offers a multispectral and multiplexing capability that is suitable for the bioimaging of inherently complicated neural systems. Fluorescence imaging probes for AD diagnosis are required to (1) be (pseudo)permeable to cross the pathologically vulnerable blood–brain barrier (BBB), (2) bind to AD-specific cells and proteins, such as Aβ and tau proteins in the brain, and (3) have absorbance and fluorescence emission spectra in the NIR window (650–1700 nm) for noninvasive imaging [122].

**Figure 8:**
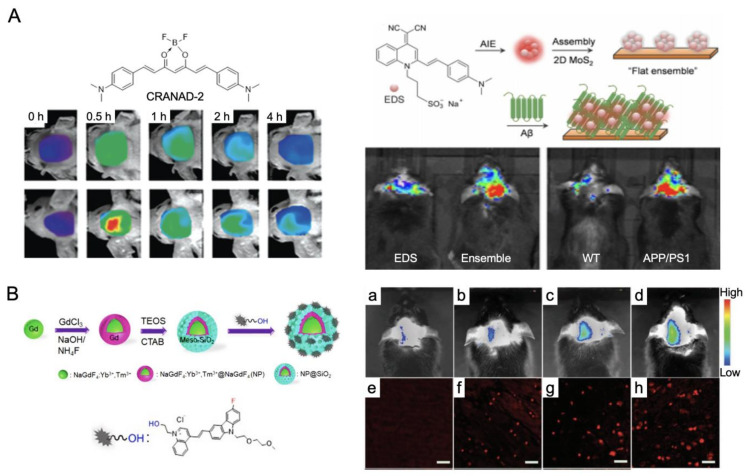
NIR fluorescence imaging for AD diagnosis. (**A**) CRANAD-2 and EDS with MoS_2_ fluorophores for the detection of Aβ fibrils in AD mice. Adapted from [128,130] with permission from Elsevier and John Wiley and Sons. (**B**) Gd-based NIR fluorescent NP@SiO2@F-SLOH for in vivo (**a**–**d**) and ex vivo (**e**–**h**) fluorescence imaging in AD mice: (**a**,**e**) from the wild type mice, (**b**,**f**) 7-month-old Tg mice, (**c**,**g**) 9-month-old Tg mice, and (**d**,**h**) 11-month-old Tg mice. Adapted from [131] with permission from John Wiley and Sons.

Figure 8 shows recent NIR fluorescence imaging using polymethine and other NIR fluorophores for AD diagnosis. As shown in Figure 8A, CRANAD-2 is a representative boron dipyrromethane (BODIPY) probe for detecting Aβ aggregates/fibrils and has a high fluorescence intensity with a blue-shifted emission [128,129,130]. CRANAD-2 self assembles in aqueous solutions, which increases its solubility, but is limited to detecting single Aβ or oligomers. On the other hand, a combination of quinoline–malononitrile AIEgen (EDS) and molybdenum disulfide (MoS_2_) enables the detection of Aβ oligomers and aggregates with a high intensity (Figure 8A). Interestingly, a recent report shows that fluorescent nanoparticles (NPs) can be used for AD theranostics. Upon surface functionalization of the mesoporous silica layer of the Gd^3+^-based NPs with an Aβ oligomer-selective cyanine dye, NP@SiO2@F-SLOH showed cell-membrane-permeability, BBB penetration, and Aβ-targeting with fast clearance from the liver and kidneys (Figure 8B) [131]. This multimodal NIR/MRI contrast agent is versatile and sensitive for the real-time detection of Aβ oligomers and plaques in AD mice. 

NIR fluorescence imaging is well-suited to the simultaneous identification of several different targets due to its ability for multichannel multispectral imaging using different irradiation wavelengths, but its noninvasive imaging has been a challenge. Using NIR fluorescent ZW800-1C (Ex @760 nm and Em @780 nm, fluorescence lifetime imaging (FLIM) enabled the noninvasive multi-detection of Aβ, tau protein, and blood vessels in transgenic mouse models of AD [132]. Hou et al. used two zwitterionic NIR fluorophores with slightly different logD and topological polar surface areas (TPSA): −3.35 and 167.18 Å^2^ for ZW800-1A and −2.80 and 157.95 Å^2^ for ZW800-1C, respectively [132]. Interestingly, the high flexibility and hydrophilicity of ZW800-1A lowered its affinity to the surface of Aβ and tau aggregates compared to ZW800-1C [133,134,135,136]. Since ZW800-1C is lifetime-sensitive, it could be used to differentiate Aβ and tau proteins along with blood vasculature for noninvasive AD pathology using FLIM tomography.

## 4. Summary and Conclusions

One underlying cause of AD is accumulated Aβ and tau proteins in the brain, which cause synaptic dysfunction, neuronal cell cycle re-entry, and neuron cell death, resulting in the shrinking of structures and veins in the brain. Functional abnormalities such as severe memory loss and impaired motor function are also significant signs for determining the presence of the disease. However, the major limitation in AD theranostics is the lack of diagnostic criteria to identify the disease early on while it is still treatable. Neuroimaging improves our understanding of AD by drawing more critical connections between different patient groups along the course of the disease, as shown by our discussion of multiple brain imaging modalities in terms of their basic principles, detection of brain alterations related to AD, and their advantages and limitations for clinical use. While a definite diagnosis of AD still requires post-mortem confirmation, brain imaging provides a supportive window for identifying and visualizing the characteristic patterns that AD entails. The existing systems hold different advantages, yet there is still a need for multimodal imaging of biomarkers to bring meaningful outcomes. Efforts will be needed at multiple ends, such as enhancing resolution or developing clinically accessible probes. Furthermore, the potential to use brain imaging for AD theranostics is expanding with the advances in machine learning and deep learning algorithms that enable the processing of an enormous amount of clinical data. With more robust validations, these data analytic methods will also be integrated with neuroimaging modalities to serve as valuable tools for clinicians to make early diagnoses and prognoses of AD.

## 5. Limitations and Future Directions

This review mainly focuses on neuroimaging modalities that measure changes at the cortical level that do not rule out the possibilities of other neurodegenerative diseases or comorbidities. No single technique reveals all answers about the pathological processes and the functional defects that come after. Therefore, current imaging methods only take up a complementary role in diagnosis at the moment. Combining structural and functional brain information clarifies the connection between pathophysiological hallmarks and neuronal functions. It has been shown that the combination of different imaging modalities helps to better classify and identify individuals in the mild symptomatic stage who may convert to severe dementia. However, most of these results come from pre-selected cohorts, and given the current evidence, future studies concerning the generalizability to broader populations are critical.

The difficulty in establishing standardized criteria for accurate staging along with the limited physiological signals associated with AD has opened up new passages for the more precise imaging of this devastating disease. The utilization of optical contrast agents in the NIR window enables the real-time noninvasive tracking of hallmark neuropathological features of AD. Though its broader application to larger patient groups may take years, the research community is making strides by developing new NIR fluorescent probes and noninvasive optical neuroimaging systems that possess more clinical utility. Since the currently available therapeutic outcomes are limited to mitigating symptoms, enhancing real-time diagnostic abilities by combining multiple approaches would lead to breakthroughs in AD diagnosis and treatment. 

## Figures and Tables

**Table 1:** Neuroimaging findings in AD patients.

Imaging Method	Principle	Main Findings	Refs.
Structural MRI	MR with hydrogen	Cerebral atrophy, ventricular enlargement	[7,8,9,10]
FDG-PET	Radioluminescence of FDG, amyloid, tau, etc.	Reduced cerebral glucose metabolism	[11,12]
fNIRS	NIR light for hemodynamics	Reduction in HbO concentration	[13]
fMRI	MR for hemodynamics	Hyper- and hypo-activation in the task-related regions	[14,15,16,17,18,19,20,21,22]
EEG	Electrical signal of brain	Altered functional connectivity pattern, slowing, decrease in complexity, alterations in microstate	[23,24,25,26,27,28,29,30]

MRI, magnetic resonance imaging; FDG-PET, fluorodeoxyglucose-positron emission tomography; fNIRS, functional near-infrared spectroscopy; fMRI, functional MRI; EEG, electroencephalography.

**Table 2:** The potential of neuroimaging methods in detecting AD.

Method	Sensitivity	Specificity	Accuracy	Pros	Cons	Refs.
MRI	80–95%	55–98%	89–97%	Spatial resolution	Temporal resolution, MR exposure	[36,105,106,107,108]
FDG-PET	43–100%	57–100%	50–100%	Clear image	FDG injection	[44,47,57,63,109]
fNIRS	82–94%	72–88%	50–90%	High speed, portability	Spatial resolution	[110,111]
fMRI	84–94%	68–91%	75–93%	Spatial resolution	Not portable/Expose MR	[78,80,112]
EEG	35–88%	82–100%	62–92%	High speed, portability	Spatial resolution	[99,101,103,113,114]

MRI, magnetic resonance imaging; FDG-PET, fluorodeoxyglucose-positron emission tomography; fNIRS, functional near-infrared spectroscopy; fMRI, functional MRI; EEG, electroencephalography.

## Data Availability

Not applicable.

## Abbreviations

AD	Alzheimer’s disease
APP	amyloid precursor protein
Aβ	amyloid-β
APOE	apolipoprotein E gene
BOLD	blood oxygenation level-dependent
BBB	blood–brain barrier
BODIPY	boron dipyrromethene
CBF	cerebral blood flow
CBV	cerebral blood volume
CTR	control subjects
CNN	convolutional neural network
DMN	default-mode network
HbR	deoxy-hemoglobin
FLIM	fluorescence lifetime imaging
HC	healthy control
HOA	healthy older adults
MCI	mild cognitive impairment
MSE	multiscale entropy
NPs	nanoparticles
HbO	oxy-hemoglobin
PCC	posterior cingulate cortex
ROI	region of interest
RSNs	resting-state networks
TPSA	topological polar surface areas
